# The transcription factor activity gradient (TAG) model: contemplating a contact-independent mechanism for enhancer–promoter communication

**DOI:** 10.1101/gad.349160.121

**Published:** 2022-01-01

**Authors:** Jonathan P. Karr, John J. Ferrie, Robert Tjian, Xavier Darzacq

**Affiliations:** 1 University of California at Berkeley, Berkeley, California 94720, USA;; 2 Howard Hughes Medical Institute, Berkeley, California 94720, USA

**Keywords:** 3D genome, coactivator, enhancer, gene regulation, p300, transcription

## Abstract

How distal *cis*-regulatory elements (e.g., enhancers) communicate with promoters remains an unresolved question of fundamental importance. Although transcription factors and cofactors are known to mediate this communication, the mechanism by which diffusible molecules relay regulatory information from one position to another along the chromosome is a biophysical puzzle—one that needs to be revisited in light of recent data that cannot easily fit into previous solutions. Here we propose a new model that diverges from the textbook enhancer–promoter looping paradigm and offer a synthesis of the literature to make a case for its plausibility, focusing on the coactivator p300.

## Abstract (precis)

In this Perspective, Karr et al. propose a new model that diverges from the textbook enhancer–promoter looping paradigm and offer a synthesis of the literature to make a case for its plausibility, focusing on the coactivator p300.

Gene regulation involves the interplay of genetically encoded circuitry with dynamic input from cellular signaling. *Cis*-regulatory elements (CREs) provide the circuitry, while transcription factors (TFs) transmit the signals. CREs’ latent potential awaits realization by TFs, while TFs’ *trans*-regulatory function depends on CREs to direct them to their target loci. The fundamental mechanism underlying this decoding of biochemical signals by TF–CRE interactions has been brought into question by recent data from live-cell microscopy experiments showing spatiotemporal dynamics at odds with current models. The unexpected results from such experiments have yet to be satisfactorily reconciled with the long-standing rules of CRE–promoter communication learned from decades of genetics, biochemistry, and genomics.

We know that CREs operate by a sequence of molecular interactions. By being enriched in TF recognition sequences, CREs recruit TFs via protein–DNA binding. CRE-bound TFs recruit other TFs as well as transcriptional cofactors—proteins or protein complexes that typically bear histone-modifying or nucleosome-remodeling enzymatic activities (Rosenfeld 2006)—via protein–protein binding. CREs thereby assemble a combination of proteins and enzymes at a particular position on the chromosome, while TFs translate DNA sequence into local enzymatic activity (acetylation, phosphorylation, methylation, etc.) via their DNA-binding and protein interaction domains, ultimately regulating RNA polymerase II (Pol II) activity at a target promoter.

How such interactions at a proximal CRE could regulate transcription is conceptually much more straightforward than at a distal CRE, which can be many kilobases, or even a megabase, upstream of or downstream from the target gene. There are at least three conceivable models by which a distal CRE could operate (Fig. 1). Model 1—“stable contact model”: By the formation of a long-lasting protein–DNA complex stabilizing a chromatin loop, the distal CRE and promoter effectively become a single compound CRE with properties that neither element possesses on its own. Model 2—“kiss-and-run model”: By transiently contacting the promoter, the CRE could deposit some material onto the promoter (be it TFs, other components of the transcriptional machinery, or post-translational modifications [PTMs] of promoter-bound proteins) that persists beyond a transient CRE–promoter contact. Model 3—“communication by diffusion model”: The CRE could communicate with the promoter in a distance-dependent manner through the diffusion of TFs activated by enzymes recruited to the CRE.

**Figure 1.**
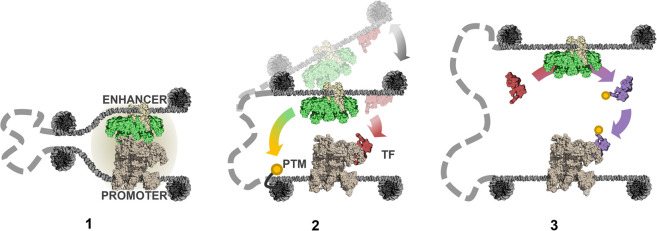
Mechanisms of enhancer–promoter communication: There are three different ways in which a distal enhancer could regulate a promoter. (1) Stable contact model: A “compound *cis*-regulatory” element is formed by a stable complex of TFs (tan), coactivators (green), and the transcriptional machinery (gray). (2) Kiss-and-run model: Upon transient contact, enhancer-bound coactivators deposit PTMs at the promoter, and TFs (red) are transferred. (3) Communication by diffusion model: TFs are activated (purple) at the enhancer and diffuse to the promoter.

Each model has distinct requirements and temporal predictions. Both models 1 and 2 fundamentally require direct contact between the CRE and promoter via DNA looping (note that by direct contact we mean an unbroken chain of molecular binding interactions, which we expect not to exceed tens of nanometers). However, whereas model 1 proposes that the promoter is active only when in contact with an enhancer and therefore necessitates persistent DNA–protein complexes tethering the CRE to the promoter, model 2 allows some memory of interaction and thus requires only transient CRE–promoter contacts at some frequency. Model 3, on the other hand, does not necessitate contact between CRE and promoter, but does have a quantitative dependence on proximity and therefore predicts that sustained contact would strengthen the effect of the CRE on the promoter.

Model 1 hearkens back to bacterial gene regulation and offers an intuitive solution to the problem of distance between enhancer (a representative class of CRE) and promoter, making it the prevailing textbook picture of *cis* regulation. It further gained popularity as the previous decade saw marked advances in chromosome conformation capture (3C) technologies, which have detected signal enrichments between enhancers and promoters (de Wit and de Laat 2012; Hsieh et al. 2020). Although such 3C signal has been widely interpreted to indicate contact, it should be remembered that 3C does not actually report on contact between genomic regions in a single live cell, but rather the probability of cross-ligation in a large population of fixed cells. That is to say, it reflects not temporal frequency but population frequency, captured under chemical cross-linking conditions, which are known to perturb both chromatin structure and TF–chromatin interactions (Teves et al. 2016; Hoffman et al. 2020). Hence, 3C data cannot be used to test different temporal predictions about the longevity of CRE–promoter contacts in vivo. Furthermore, 3C methods cannot discern whether CRE–promoter contact is at all necessary for transcription in single cells, so they cannot verify or falsify any of the three models.

To test temporal predictions, microscopy experiments are required to fill in the gaps in our knowledge concerning CRE–TF interaction dynamics and CRE–promoter distances in single cells. Such experiments have consistently surprised us by yielding results dissonant with expectations from models 1 and 2, and even incompatible with their basic requirements. Single-particle tracking experiments measuring the diffusion of nuclear proteins have documented fleeting lifetimes of TF–chromatin interactions, from hundreds of milliseconds to several seconds (for review, see Liu and Tjian 2018). Such rapid dynamics are obviously difficult to reconcile with model 1. Moreover, recent experiments measuring both distal CRE–promoter distances and promoter activity in single cells (for a review of methods, see Brandão et al. 2021) have not supported either model 1 or 2. For enhancers removed by scores to hundreds of kilobases, promoter activity was shown to have dependence on proximity (∼350 nm) in one case (Chen et al. 2018), an anticorrelation with proximity in another case (Benabdallah et al. 2019), and no correlation with closer proximity in another case (Alexander et al. 2019). A FISH method able to probe many interactions in a single cell likewise saw weak correlation between contact and activity in some enhancer–promoter pairs, and no correlation in others (Mateo et al. 2019). The basic requirement for contact in models 1 and 2 is not satisfied in these instances. Moreover, several experiments perturbing proteins involved in global 3D genome organization have shown at most mild effects on transcription output despite profound losses of 3C signal (Nora et al. 2017; Rao et al. 2017; Hansen et al. 2019; Luan et al. 2021). It is theoretically possible to reconcile the kiss-and-run model with such weak or absent temporal correlations of promoter activity and enhancer–promoter proximity only if it is assumed that there is an enduring “memory” of interaction such that each contact contributes to an accumulating signal at the promoter, whether in the form of proteins or of protein PTMs (Xiao et al. 2021). We find that to be a dubious assumption given the transience of protein–DNA interactions and of PTM lifetimes and note that it still strictly requires contact; indeed, the frequency of contact must be inversely proportional to the length of memory. Last, because the modeling from Xiao et al. (2021) was done in arbitrary time, we cannot know whether the infrequency of contacts seen in recent microscopy experiments is reconcilable with the proposed theory.

We are led then to one of two conclusions: Either the recent studies from multiple groups were technically unable to observe the phenomenon of enhancer–promoter looping in single cells, or it is much rarer and of lesser regulatory importance than has been supposed. Therefore, although contact-dependent models have not been entirely disproven, to consider an alternative model may nonetheless be warranted by the new evidence at hand, and could prove useful in instigating discussion of a broader range of mechanisms. The “communication by diffusion” model has largely been disregarded because it poses a fundamental problem believed to be irreconcilable with the physics of diffusion; namely, it depends on TF molecules visiting the CRE and subsequently binding promoters in *cis* with a higher probability than other DNA elements in the nucleus. However, we have conceived of what seems to be a plausible mechanism for CRE–promoter communication via diffusion. We call it the TF activity gradient (TAG) model, since it consists of CRE-associated enzymes modifying TFs to create local 3D gradients of chemical signals. We find the TAG model attractive in that it derives naturally from longstanding but previously unconsolidated observations, and it grounds CRE–promoter communication in exquisitely regulable enzymology, without relying on the more topologically constrained and convoluted process of intrachromosomal contact.

## A new model of CRE–promoter communication

The road to the TAG model began with recognizing that the substrate ranges of so-called histone-modifying enzymes (or “epigenetic writers”) are actually not restricted to histone substrates, but invariably include TFs (Rosenfeld 2006; Biggar and Li 2015; Narita et al. 2019). Hence, positions along the chromosome enriched for histone PTMs represent likely sites for enhanced TF modification. This suggests the intriguing possibility that CREs, which bear a significant number and diversity of histone modifications, could act as inducible platforms for catalytic modification of TFs. CREs could thereby serve a function analogous to that of scaffold and targeting proteins for signaling kinases; namely, to bring together promiscuous enzymes with specific substrates in a regulable and localized manner, or to coordinate signal relays by clustering different enzymes in a pathway together (Langeberg and Scott 2015).

Take for example the transcriptional coactivator and lysine acetyltransferase CBP/p300 (referred to here as just p300), which has long been appreciated as a central player in gene regulation. Levels of histone H3K27 acetylation, its signature chromatin mark, at CREs have been used to predict nearby promoter activity (Karlic et al. 2010; Fulco et al. 2019), and recruiting the catalytic core of p300 to enhancers via a dCas9 fusion is sufficient to activate target promoters (Hilton et al. 2015). However, the precise mechanism by which p300 regulates transcription has remained unclear. Some puzzling reports have indicated that although p300 catalytic activity is necessary for enhancer function (Raisner et al. 2018), H3K27 acetylation is not (Catarino and Stark 2018; Zhang et al. 2020). This discrepancy could be resolved if nonhistone substrates were its functional targets and the histone mark only a collateral effect of its local activity. Since H3K27ac has never been causally linked to transcription and p300 does indeed exhibit a promiscuous substrate range, including scores of TFs whose regulatory activities are often modulated by acetylation (Dancy and Cole 2015; Weinert et al. 2018), we entertain and expand on this possibility here. (It should be noted that p300 was dubbed a histone acetyltransferase only because of the historical coincidence that histones were its first discovered substrates, not because they were demonstrated to be its specific or functional substrates.)

**Figure 2.**
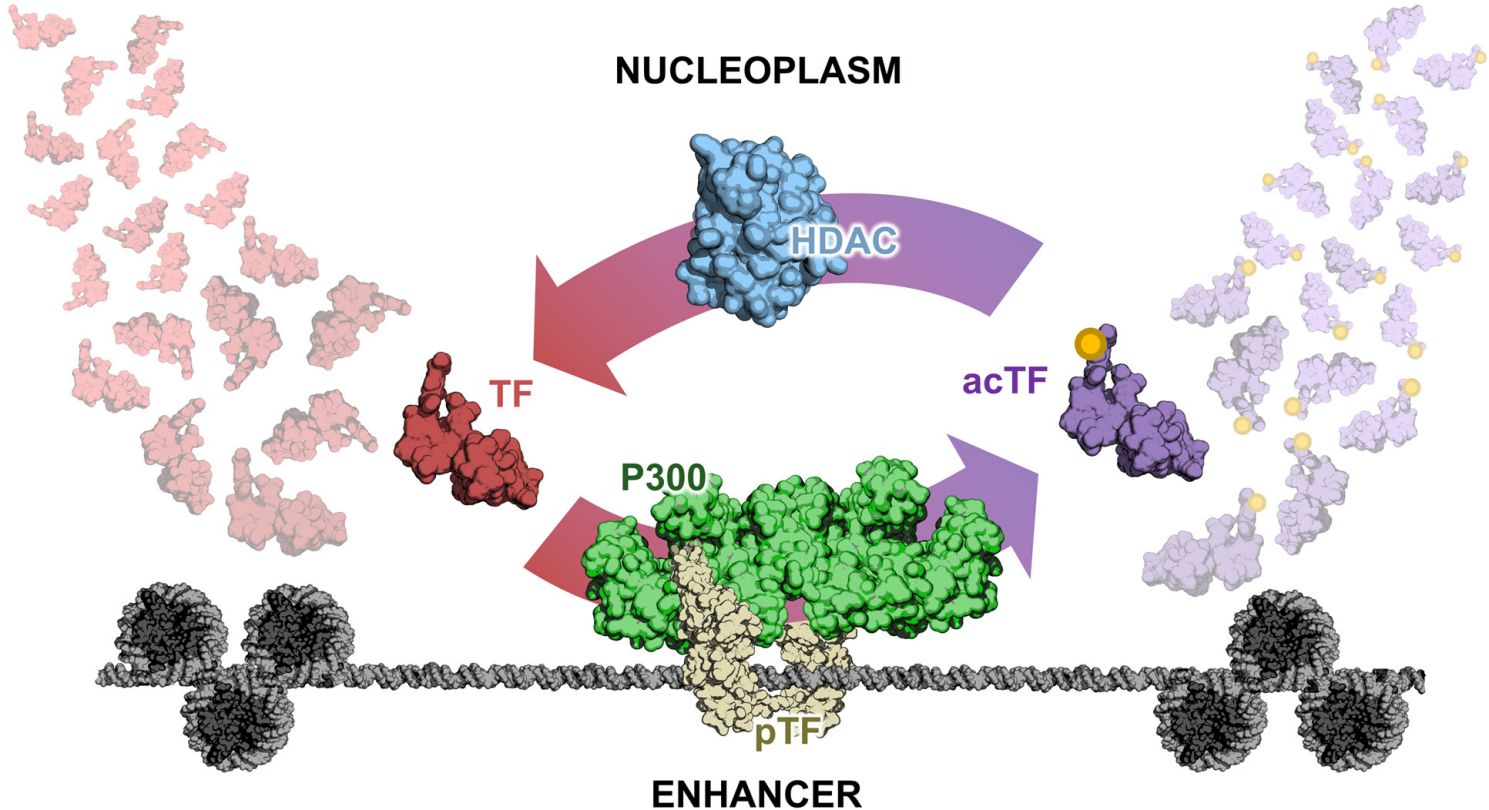
Picturing the enhancer as a point source of acetylated TFs. An unmodified TF (red) contacts an enhancer bearing activated p300 (green) recruited by a dimeric transcription factor (tan). The acetylated transcription factor (purple) departs from the enhancer and is recycled by being rapidly deacetylated in the nucleoplasm by abundant histone deacetylases (blue).

If TFs are acetylated at p300-bound enhancers (Fig. 2), the result will be spatially heterogeneous distributions of chemical signals in the form of acetylated TFs (ac-TFs). To see this, let us consider what happens immediately after a TF is acetylated by p300. As time passes, the further the ac-TF diffuses from p300, and the more likely it is to encounter a deacetylase—an abundant and ubiquitous class of enzymes also named after their histone substrates (“HDACs”) even though they have many others (Glozak et al. 2005). As a result, p300 at a CRE becomes the point source of a concentration gradient of ac-TF. If the point source is free to diffuse throughout the nucleus, local concentration gradients will not form. However, if active p300 is bound to chromatin while the TFs remain diffusible, then a gradient will arise centered on the enzyme-bound chromatin region—i.e., an enhancer. Consequently, a promoter proximal to an enhancer is far more likely to encounter an ac-TF than a promoter distant in 3D space.

Note that at equilibrium, in the absence of PTM deposition, there cannot be stable gradients of TFs arising from CREs. The existence of a nearby binding site for a TF in no way enhances its equilibrium occupancy at the promoter (in fact, the more competing sites there are, the lesser the occupancy will be at a given promoter). Even if we consider that the two elements contact one another at some rate (as in model 2), the equilibrium remains unchanged by the contact. Although it is true that upon contact a CRE-bound TF would have a higher likelihood of unbinding the CRE and binding the promoter, reciprocally a promoter-bound TF would be more likely to unbind the promoter and bind the CRE. Therefore, without invoking nonequilibrium processes, the only way in which a distal binding site could increase the TF's occupancy at the promoter is if simultaneous binding at both sites through DNA looping cooperatively strengthened the TF–promoter interaction (as in model 1).

It is the presence of an enzymatically generated gradient of activity that overcomes the major difficulty with model 3 alluded to before: TAG requires proximity (acts in *cis*) but does not depend on contact between enhancer and promoter (can operate over hundreds of nanometers in 3D space, accounting for very distal CREs). Enzymatic deposition of PTMs on TFs at a chromatin site would give rise to such a gradient for two reasons: (1) The volume through which the signal spreads increases cubically with radial distance from the enhancer, rapidly diluting the concentration of ac-TFs, and (2) an approximately constant rate of deacetylation due to abundant HDACs leads to an exponential decay profile at steady state (similar to morphogen gradients). The *cis* relationship between enhancer and promoter is therefore maintained not by the two *cis* elements physically associating, but by the biophysical and biochemical limitations on the extent of the signal diffusing from the enhancer.

Unlike the kiss-and-run model, TAG does not rely on a coordinated, vectorial transfer of material from enhancer to promoter; it is mediated by the random motions of ac-TFs emanating from the enhancer, with a small subset finding the promoter. TAG therefore has an inherent inefficiency: Since each ac-TF has a low probability of finding its target promoter, enhancer-bound p300 must modify many TFs over time. This inefficiency is “paid for” not in the currency of acetyl-CoA, which gets regenerated in situ by nuclear synthases, but in the ATP those synthases consume (Sivanand et al. 2018). The probability of the ac-TF finding its target can also be increased in two ways: by altering the mode of its diffusion or by effectively increasing its target's size (Izeddin et al. 2014). If the ac-TF undergoes subdiffusion that causes it to more densely sample its immediate environment (for instance, by sliding or hopping on chromatin), it becomes much more likely to find a local target (Bénichou et al. 2010). If at the target promoter there is even a transient hub of locally concentrated proteins (as have been observed in various systems) (Chen et al. 2014; Cho et al. 2018; Chong et al. 2018; Liu et al. 2014; Mir et al. 2017; Tsai et al. 2017) with affinity for the ac-TF, this could multiply the probability of each TF finding the promoter and thereby sensitize the promoter to the enhancer. Isotropic diffusion from the enhancer would result in a broad (if confined) range of enhancer influence and thereby would allow for one enhancer to regulate multiple promoters, which is a documented property of enhancers (Fukaya et al. 2016; Symmons et al. 2016).

Based on published findings, we propose the following TAG-based mechanism for p300-mediated regulation of transcription occurring at a generic enhancer–promoter pair: (1) p300 is first recruited to the enhancer by a sequence-specific DNA-binding TF. (2) An allosteric regulator binds and activates p300. (3) Active p300 acetylates nearby substrates, including both histones and TFs bound to the enhancer. (4) Subsequently acetylated TFs (with typical residence times of a few seconds) disengage from the enhancer and diffuse outward. (5) When ac-TFs reach a target promoter, they increase transcriptional output. (6) Diffusing ac-TFs are deacetylated at a high rate, limiting the spatial range of their action. We now walk through this hypothetical realization of the TAG model and present some supporting evidence for each step while pointing out some of the unknowns.

1. p300 is recruited to the enhancer. p300 is such a pervasive enhancer-bound coactivator that its ChIP-seq binding profile is routinely used to identify enhancers (Heintzman et al. 2007; Visel et al. 2009). Not much is known about the recruitment mechanism of p300, except that it depends on sequence-specific TFs and is subject to competition, as the number of p300 molecules per cell (∼7000) is on par with the number of active enhancers (Giordano and Avantaggiati 1999; Gillespie et al. 2020). Thus, with respect to p300, the nucleus is indeed a heterogeneous landscape, with enhancers being rare landing pads to enable and direct its enzymatic activity.
2. An allosteric regulator of p300 binds the enhancer and activates p300. The presence of H3K27ac, and not p300, differentiates active from poised enhancers (Creyghton et al. 2010; Rada-Iglesias et al. 2011), indicating that at least in some instances recruitment and activation of the enzyme are separable events. Biochemical and structural studies have uncovered allosteric regulation of the catalytic activity of p300. Similarly to how some kinases dimerize and undergo *trans*-autophosphorylation, p300 is activated by *trans*-autoacetylation (Thompson et al. 2004). Dimeric (and often phosphorylated) TFs mediate this *trans*-autoacetylation by bringing together two p300 molecules (Ortega et al. 2018). This mechanism integrates cellular signaling in that many cytosolic signaling pathways lead to dimerization and nuclear translocation of TFs to affect gene expression (Whitmarsh and Davis 2000). For many inducible TFs, signaling-mediated oligomerization has been shown to recruit active p300 at target enhancers and promoters (Wathelet et al. 1998; Mayr and Montminy 2001; Schuringa et al. 2001; Freedman et al. 2002; Zhong et al. 2002; Major et al. 2004; Simonsson et al. 2006; Kaypee et al. 2018). Where these TF–p300 complexes bind is cell type-specific and depends on the prior establishment of accessible CREs by pioneer factors (Vahedi et al. 2012).
3. Active p300 acetylates TFs bound at the enhancer. To our knowledge, it has never been investigated whether p300 acetylates nonhistone substrates on chromatin or throughout the nucleoplasm. However, it was found that p300 substrate specificity is higher in vivo than in vitro (Weinert et al. 2018), indicating that its cellular context is important for restraining the action of this highly promiscuous enzyme. Additionally, the fact that known TF substrates of p300 have ChIP-seq signals at chromatin loci enriched for H3K27ac suggests that at least some TF acetylation may occur on select chromatin sites. Furthermore, for many TFs, the dimerization that is required for p300 activation is typically required for their DNA binding as well, with nuclear receptors being an iconic example (Khorasanizadeh and Rastinejad 2001). Furthermore, in the case of the TF p53, consensus sequence DNA acts as an allosteric ligand to promote acetylation by p300 through exposure of the acetylation motif (Dornan et al. 2003; Češková et al. 2006), suggesting that p300 substrate specificity can also be mediated by the proximal DNA. Multiple lines of evidence therefore point to chromatin as a likely site of p300's TF-modifying activity.
4. Acetylated TFs disengage from the enhancer and diffuse outward. Within seconds of landing at a CRE and possibly being acetylated, a TF will unbind and continue diffusing (Liu and Tjian 2018). Some acetyl-lysines are bound by bromodomains (Zaware and Zhou 2019), which in turn can change TF–chromatin interactions and thus alter TF occupancy at target sites (Lamonica et al. 2011). Other acetyl-lysines induce conformational changes to expose new surfaces for interaction or (de)stabilize existing ones (Liu et al. 2019). Acetylation can also weaken protein–DNA interactions by neutralizing the positive charge on a phosphate-interacting lysine (Louphrasitthiphol et al. 2020). Consequently, it is plausible that an ac-TF will exhibit modes of diffusion distinct from those of the unmodified TF.
5. When acetylated TFs reach a target promoter, they increase transcriptional output. Increased transcriptional activity upon acetylation has been demonstrated for various TFs (Boyes et al. 1998; Polesskaya et al. 2000; Soutoglou et al. 2000; Tomita et al. 2000; Zhang et al. 2001; Chen and Greene 2003; Lévy et al. 2004; Sun et al. 2009; Wang et al. 2011, 2012; Zhuang 2013; Reed and Quelle 2015), although the mechanism is not clear in most cases. One notable mechanism is through interaction with bromodomain-containing protein BRD4 to recruit P-TEFb to promoters and phosphorylate Pol II (Narita et al. 2021). However, it is possible that the mechanisms at play are as diverse as the TFs being acetylated. Acetylation, like many PTMs, need not have one outcome but rather provides a regulatory switch that can modulate protein activity positively or negatively. Such flexibility may be key to achieving combinatorial specificity and complexity, allowing the same enzyme to exert positive or negative regulatory effects on transcription depending on what substrates are present.
6. Acetylated TFs are deacetylated at a high rate, limiting the spatial range of their action. Once modified at a CRE, an ac-TF has a lifetime dictated by the abundance and activity of deacetylases, of which there are 18 varieties in humans (Seto and Yoshida 2014). Recently, a proteomics study of erythropoiesis documented what the investigators described as a “vast quantitative imbalance” between the number of HDAC molecules (in the hundreds of thousands) compared with that of p300 (at <10,000) (Gillespie et al. 2020). Although live-cell imaging of HDACs is lacking, by immunofluorescence they are predominantly nuclear and homogeneously dispersed (e.g., Ververis and Karagiannis 2012). Evidently, the mammalian nucleus has evolved to keep global acetylation levels tightly controlled, which we speculate is to enable local signaling. Hence, we predict the extent of ac-TF signal to be exquisitely spatially restricted around the CRE point source.

## Conclusions and outlook

The TAG model offers a novel solution to the two key problems of CRE–promoter communication: What signal is transmitted from distal CREs to promoters, and by what mechanism does the transmission occur? We envision that the signal could be cofactor-deposited PTMs on TFs that alter their *trans*-activating/repressing potential, and the mechanism of communication is an enzymatically time-limited diffusion from the CRE point source. That TFs will be modified at CREs is a logical consequence of two established observations: CREs are TF-binding hotspots replete with histone signatures of cofactor activity, and those TFs are known substrates of cofactor enzymes. Chromatin-associated enzymatic activities in turn result in spatial heterogeneity of chemical signals given diffusible substrates and the profusion of demodifying enzymes that limit the lifetime of the PTM. The requirement for distal CRE–promoter proximity is therefore met without necessitating direct or sustained contact between the two elements, and the parameter that tunes promoter activity is the PTM–TF flux from nearby CREs, not the frequency or stability of direct contact between two chromosomal regions.

Although it has long been known that CREs are hotspots of cofactor activity, the chemical ramifications of PTM-depositing enzymes being recruited to defined positions along the chromosome have largely gone unexplored. Perhaps this is partly due to the fact that TF–CRE binding interactions are widely treated as mere equilibrium-driven associations, instead of a platform for nonequilibrium modification of the TFs by CRE-associated enzymes, even though it has been appreciated since almost the beginning of the enhancer field that covalent modification of TFs was key to their regulation (Maniatis et al. 1987). Given that PTM-depositing enzymes are enriched at CREs, where we now know TFs rapidly bind and unbind within seconds, and that so-called histone-modifying enzymes are vastly outnumbered by their corresponding demodifying enzymes (Gillespie et al. 2020), we propose that CREs function as local “reactors” to generate tunable concentration gradients of modified and activated TFs that diffuse to nearby binding sites to regulate target promoters.

Since PTMs have profound effects on TF interactions and function (Filtz et al. 2014), the ability to locally and transiently concentrate them provides a mechanism for precise control of gene regulation. If, for instance, a TF is only 10% active in its unmodified state, an increase in its nuclear concentration will have relatively mild effects on target gene transcription except in regions where it acquires its activating PTM. Consequently, TF-responsive promoters with nearby CREs able to deposit the PTM will be much more activated than target promoters lacking such CREs. Conversely, a TF may activate transcription when unmodified but repress transcription when modified. The ensemble of active CREs and their associated enzymes would therefore determine the global transcriptional changes in response to the presence of a TF. Consistent with this notion, it has been shown that inducible TFs bind pre-existing CREs that are made accessible by lineage-determining TFs (Heinz et al. 2015), that proximal promoter activity is correlated with recruitment of enzymatic coactivators to such pre-existing CREs (Vahedi et al. 2012), and that DNA accessibility of CREs is just as determinative of TF occupancy as the presence of TF recognition sequences (Li et al. 2011).

Different TFs therefore play different roles in the TAG model. Certain TFs are necessary to define which CREs are accessible, other TFs bind the accessible CREs to recruit cofactors, and perhaps still other TFs are the substrates of those cofactors that will carry the signal to nearby promoters. Importantly, specificity is attained by sequence-specific protein–DNA and selective protein–protein interactions at each of these steps. So-called master regulators or pioneer factors find recognition sequences to open up a subset of CREs (Iwafuchi-Doi and Zaret 2016). Which TFs bind available CREs is determined by their specific DNA affinity (Spitz and Furlong 2012), and what proximal genes respond to an active CRE depends on the PTM–TF binding at the promoter—whether indirectly through protein–protein interactions with promoter-bound TFs or directly through the PTM–TF binding the DNA. Other mechanisms can also be at play due to the number of tunable parameters in the system. For example, in some rare instances, disruption of TAD boundaries can lead to ectopic expression of a promoter (e.g., Lupiáñez et al. 2015), indicating that at certain loci in specific cell types, part of the CRE–promoter specificity can be influenced by the 3D organization of local chromatin. Hence, although the TAG model is not explicitly concerned with the question of specificity (that is, why a CRE affects certain promoters in its vicinity and not others), it nevertheless hints at a possible path to specificity mediated, satisfyingly, by sequence-specific DNA-binding proteins and their protein–protein interactions.

Although it is conceptually helpful to imagine one PTM–TF communicating from a CRE to a promoter, it is likely that multiple protein species contribute to the PTM gradient, some of which may not be sequence-specific TFs. Because some active enhancers are transcribed, we can surmise that much of the transcriptional machinery is being recruited to them; components of that machinery that are modified should also give rise to a local gradient due to diffusion from the CRE and deactivation by repressing enzymes. For instance, general transcription factors, the elongation factor P-TEFb, and Pol II are all acetylated by p300 (Imhof et al. 1997; Cho et al. 2009; Schröder et al. 2013). Of these, P-TEFb is an especially intriguing candidate in that it is broadly required for Pol II transcription, depends on p300 for activation, exhibits subdiffusion, and can be rapidly inactivated by the 7SK complex (Cho et al. 2009; Izeddin et al. 2014; C. Quaresma et al. 2016). The TF c-Myc may also be an important CRE–promoter relay molecule, in that it generally amplifies transcription from active promoters (Lin et al. 2012; Nie et al. 2012), and is known to be acetylated and activated by p300 (Vervoorts et al. 2003; Faiola et al. 2005).

Since TAG depends on PTM of diffusible molecules, what role, if any, do histone modifications play in this model? It should be acknowledged that the respective contributions of histone PTMs and TF PTMs to gene regulation are challenging to disentangle; the fact that the same enzymes are likely modifying both substrates at the same locations makes correlations abound. However, it is difficult to imagine how PTMs at a CRE could participate in regulation of a distal promoter. It would seem that in order for histone modifications to be directly involved in CRE–promoter communication, a CRE-bound enzyme would need to modify promoter-bound histones, necessitating at least transient contact between these two elements. Even if it is granted that CRE–promoter contact is a requisite for regulation, there is no obvious mechanism for achieving specificity. If an active CRE-bound enzyme modifies any histones it contacts, how are promoters selectively modified over intergenic regions, or some promoters activated while others in the vicinity are not? Moreover, as noted above, the histone PTMs would need to have a lifetime much greater than that of the CRE–promoter contact, which is difficult to imagine given the pervasiveness of HDACs. This difficulty could perhaps be surmounted if a CRE–promoter contact initiates a feed-forward loop in which a histone mark deposited by the CRE-bound enzymes recruits more histone-modifying enzymes to the promoter. However, such a system seems alarmingly unregulable as well as unspecific. We therefore posit that histone modifications may not play an instructive or causal role in transcription initiation, but may rather have a permissive role in maintaining CREs in a particular state; for instance, by stabilizing TF-recruited and -activated enzymatic cofactors on the chromatin, or creating local chromatin landscapes that influence TF–chromatin interactions and therefore TF diffusion.

Another aspect of CRE–promoter communication that has garnered much attention for decades but has received little comment here is the role of boundary elements or insulators. These elements are described by three major features: In an orientation-sensitive manner they can prevent an enhancer from communicating with a promoter, they delineate boundaries between active and repressive chromatin marks, and they are associated with TAD boundaries in 3C assays. TADs have also been shown to correspond to domains over which an enhancer can activate a promoter (Anderson et al. 2014; Symmons et al. 2016). Whereas *Drosophila* have several known insulator-binding proteins, the only one that has been characterized in mammalian cells is CTCF. Since depletion of CTCF has very mild effects on transcription, both in number of genes affected and in the magnitude of that effect (Hsieh et al. 2021), insulators were not a focus here. However, generally speaking, the TAG model accounts for boundary elements in that TAD organization will inform 3D distances, which will then affect the ability of a CRE to influence a promoter; that is, if a topological boundary moves a promoter outside an enhancer's gradient of activity, it should effectively insulate the two elements in a seemingly stepwise fashion. Within a TAD, however, TAG would predict that there would be a graded effect as a function of enhancer–promoter distance, as has been documented at least once (Zuin et al. 2021). The less compact the chromatin is, the more dramatic this effect should be, which could explain why the loop-extruding cohesin complex is necessary for distal but not proximal enhancers (Kane et al. 2021). The insulator-defined boundaries of histone modifications also suggests that between TADs, protein–chromatin interactions may differ and could change the local diffusion dynamics of TFs (e.g., if one TAD has more accessible DNA than a neighboring TAD and thereby better retains the TF) (cf. McSwiggen et al. 2019).

Various groups, including our own, have observed that at the sub-TAD level, a remarkable pattern of enhancer–promoter cross-ligation is visible by Hi-C and Micro-C (Schoenfelder and Fraser 2019; Hsieh et al. 2020). Additionally, the Engreitz laboratory (Fulco et al. 2019) was able to use Hi-C signal enrichments in a powerful strategy to predict functional enhancer–promoter pairs. Their model includes only a few parameters: read counts of H3K27ac ChIP-seq and DNase hypersensitivity (which they collectively dub “activity”) and “contact” (Hi-C enrichment). Impressively, the product of activity and contact normalized against the surrounding 5-Mb region correlated with quantitative effects on gene expression with 70% precision at 70% recall. The investigators further make an intriguing observation that Hi-C contacts can be replaced by linear distance with little damage to the power of the model. The implication of this is that higher levels of enzymatic activity can compensate for greater distances between enhancers and promoters—a result that is easily rationalized by TAG: at greater distances from the enhancer, the greater the fold decrease in the ac-TF gradient, so the greater the initial signal (activity) must be to compensate. We therefore propose that a more conservative interpretation of 3C data may also be the more biologically relevant: 3C signal may actually reflect 3D proximity, not contact. We also suggest that the ability of higher activity to compensate for greater distance is more easily explained by TAG than by contact-dependent models.

Direct demonstration of the TAG model would require tracking locally deposited and exceedingly transient chemical modifications of diffusing proteins in live cells. Although local gradients of a small molecule in vivo have been measured (Bock et al. 2021), detecting a PTM gradient would require new technological developments. Nevertheless, TAG makes some predictions that differentiate it from other models. First, CRE–promoter communication should be dependent on distance but not on contact. Unfortunately, with the spatiotemporal resolutions of techniques currently available, these two parameters are difficult to tease apart. Second, TAG predicts that the regulatory effect of a cofactor—whether activating or repressive—will depend on the TF modified. This prediction differentiates TAG from histone-centric models in that a histone modification would be predicted to have the same effect at different CREs (unless, as the histone code hypothesis proposed, histone PTMs occur in complex combinations that have emergent regulatory properties, but the high redundancy of histone marks suggests this mechanism is not likely widely used) (Rando 2012). Furthermore, TAG predicts that gain-of-function mutations of cofactor-modified residues of TFs should bypass the dependence on CREs at target promoters where the PTM–TF is sufficient for activation or repression.

While it requires substantial experimental validation, the model presented here provides one plausible alternative to DNA-looping models for how TF inputs are dynamically processed by genetic circuitry into transcriptional outputs: DNA sequence determines local enzymatic activity, which in turn dictates the regulatory function of diffusible TFs at proximal promoters. Such a mechanism harnesses the properties of the nucleus that make it qualitatively different from a test tube—spatial confinement and heterogeneous distributions of molecules—allowing local effects and not just global parameters (e.g., TF concentration) to have profound regulatory impact. While posing challenges to current experimental techniques, such a system would afford exquisite control of gene expression via precise DNA targeted enzymatic control of promoter microenvironments. We conjecture that CREs evolved to do just that—to generate neighborhoods of chemical signals in which TFs can play TAG.
